# How to use lactate levels to guide septic shock resuscitation: an update

**DOI:** 10.62675/2965-2774.20260083

**Published:** 2026-07-07

**Authors:** Jan Bakker, Mervyn Singer

**Affiliations:** 1 Erasmus MC University Medical Center Department of Intensive Care Adults Rotterdam Netherlands Department of Intensive Care Adults, Erasmus MC University Medical Center - Rotterdam, Netherlands.; 2 University College London Bloomsbury Institute of Intensive Care Medicine London United Kingdom Bloomsbury Institute of Intensive Care Medicine, University College London - London, United Kingdom.

## INTRODUCTION

Although the degree of hyperlactatemia in critically ill patients is associated with an increasing risk of mortality,^(1)^ it is perhaps better considered an indicator of body stress. The traditional belief that hyperlactatemia was a direct surrogate of tissue hypoxia led to hemodynamic interventions focusing on normalizing lactate levels as a marker of achieving adequate tissue oxygenation. However, a greater appreciation of the mechanisms underlying elevated lactate levels has tempered enthusiasm for a primary lactate-targeted approach and emphasized the need to consider hyperlactatemia in the context of other physiological and biochemical variables and the underlying clinical condition. Recent studies have added new insights into the relationship between lactate levels, interventions, and outcomes, mandating a different view of how lactate levels should be used in clinical practice.

## LACTATE METABOLISM IN HEALTH AND DISEASE

**Figure 1:**
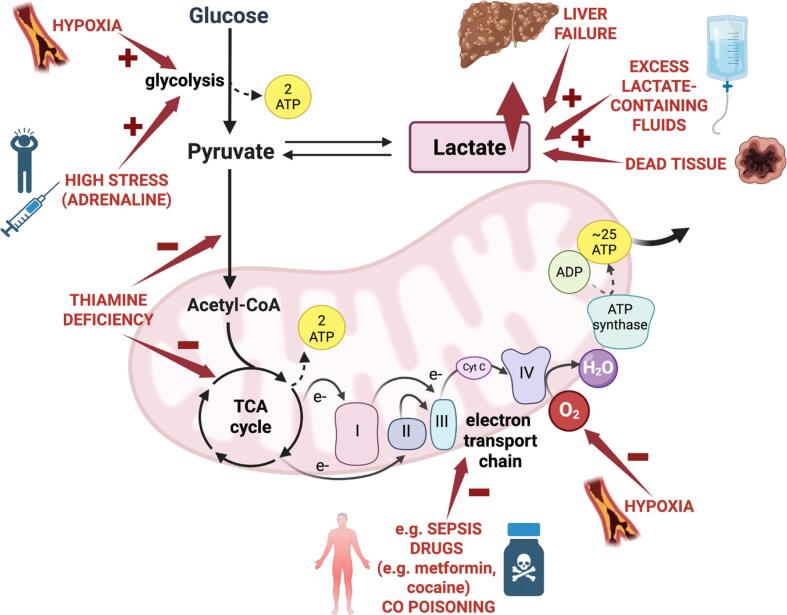
Determinants of lactate levels in critically ill patients.

Glucose, fat, protein, ketones, and lactate are all energy substrates utilized variably by different cell types in health and disease. These substrates are metabolized to feed into the mitochondrial Krebs (citric acid) cycle, which, in turn, transfers electrons to the electron transport chain (ETC) that drives the generation of adenosine triphosphate (ATP) (Figure 1). This process, oxidative phosphorylation, depends on the presence of oxygen, the terminal electron acceptor of the ETC. For every mole of glucose, two ATP molecules are generated by glycolysis, two by the Krebs cycle, and approximately 25 by the ETC. This highlights the dependency of all cell types on functioning mitochondria to fuel their metabolic processes (except erythrocytes, as they don't contain mitochondria).

ATP - adenosine triphosphate; Acetyl-CoA - acetyl-coenzyme A; TCA - tricarboxylic acid; ADP - adenosine diphosphate.

In the context of tissue hypoxia, insufficient oxygen prevents the mitochondrial ETC from generating aerobic ATP. Accordingly, there is a build-up of Krebs cycle metabolites. This results in decreased utilization of pyruvate, the end-product of glycolysis, which is metabolized by pyruvate dehydrogenase to acetyl-coenzyme A (acetyl-CoA), its entry point into the Krebs cycle. As cytosolic pyruvate is in equilibrium with lactate, the lactate level rises. This increase in lactate production is exacerbated further by a rapid upregulation of glycolysis, which produces more ATP anaerobically to partially compensate for the drop in aerobic ATP. In sepsis and other inflammatory conditions, even in the absence of tissue hypoxia, other derangements occur that increase lactate. Such derangements include inhibition or damage to enzymes within the ETC and Krebs cycle, and inhibition of pyruvate dehydrogenase.

A further important contributor to hyperlactatemia is the provision of lactate from skeletal muscle to other body organs as an energy-rich substrate that can be converted back to pyruvate. This represents an important adaptive process during times of stress to feed other cells, including cardiomyocytes, neurons, and renal cortical cells.^(2)^ Significant amounts of lactate are released from muscle through adrenaline-stimulated aerobic glycolysis and activation of the membrane Na^+^/K+ pump.^(3)^ This was elegantly demonstrated by Levy et al. in patients with septic shock.^(4)^

Other non-hypoxic causes of hyperlactatemia include, for example, exogenous adrenaline administration, use of drugs that inhibit oxidative phosphorylation, e.g., metformin (metformin-associated lactic acidosis [MALA]) or high doses of propofol (propofol infusion syndrome), thiamine deficiency, which affects glycolysis and the Krebs cycle, impaired lactate clearance, and significant infusions of lactate-containing fluids, especially in the latter condition. In health, the liver and kidneys are the main metabolizers of lactate, recycling it back to glucose via the Cori cycle. An acutely failing liver and/or kidneys can result in increased lactate levels.

## RELEVANCE OF HYPERLACTATEMIA

Increased lactate levels are associated with increased morbidity and mortality in both prehospital care and in patients admitted to the hospital with various clinical conditions.^(5,6)^ Higher initial levels, higher peak levels, and protracted normalization of lactate levels are all poor prognostic risk factors.^(7)^ This increased mortality risk even begins within the normal lactate range, but rises exponentially as lactate levels rise.^(5,8)^ Using point-of-care devices, blood lactate levels can be measured within minutes, so an at-risk patient can be quickly identified. However, the underlying diagnosis may not be immediately apparent unless there is a clear clinical picture, such as seizures, overdose, massive hemorrhage, or cardiac arrest. It is therefore crucial to establish a clinical context to identify the cause and institute effective treatment that may benefit the patient.^(9)^ It is also important to regularly re-evaluate the need for this treatment, as excessive treatment may be associated with harm.^(10)^

## TISSUE PERFUSION AND LACTATE LEVELS

Poor perfusion can lead to a critically low tissue oxygen delivery, which may result in organ dysfunction. Prompt resuscitation is a priority underlined in many guidelines, as delayed restoration of an adequate tissue oxygen delivery is strongly related to morbidity and mortality.^(5)^ In experimental studies, a progressive decrease in global blood flow eventually results in a state where oxygen consumption is dependent on oxygen delivery (supply dependency), causing a sharp increase in blood lactate levels. In such models where cardiac output is decreased, such as tamponade or hemorrhage, restoration of global blood flow usually results in rapid normalization of blood lactate levels. However, in sepsis models, restoration of global blood flow often fails to correct hyperlactatemia. This is likely related to the effects of sepsis on glucose metabolism, pathologic changes in endothelial function that limit microcirculatory perfusion and do not fully respond to improvements in the microcirculation, and mitochondrial dysfunction.^(8,11–13)^ Early normalization of lactate levels following initial resuscitation reflects restoration of tissue perfusion.^(14)^ In an emergency room study, normalization of capillary refill time (CRT) with fluid resuscitation, as a marker of adequate peripheral perfusion, was associated with a decrease in lactate levels and improved outcomes.^(15)^ In patients in whom CRT did not normalize, lactate levels were very high (> 10mmol/L); this was associated with a 7-fold higher mortality compared to patients with normal CRT (67% *versus* 9%, respectively). These findings underscore the importance of not using lactate levels in isolation but rather using them in conjunction with other variables.^(1,9)^

## TREATMENT OF PATIENTS WITH INCREASED LACTATE

When increased lactate levels are considered a marker of tissue hypoperfusion, interventions to increase blood flow and perfusion pressure are a logical first step and are generally associated with better outcomes. However, caution should still be applied. The ANDROMEDA-SHOCK study in patients with septic shock compared early resuscitation based on decreasing (normalizing) lactate levels against CRT normalization. Although no overall significant outcome difference was seen between the groups, post-hoc analysis identified a negative impact in patients in the lactate arm in whom the lactate level was not normalized (or had a decrease < 20%) after the first 2 hours of treatment, but who were subjected to continued protocolized treatment aimed at improving tissue perfusion.^(10)^ An approach targeting not only a decrease in blood lactate levels but also correcting abnormal flow-related variables, such as CRT or veno-arterial partial pressure of carbon dioxide (PCO_2_) difference, may be useful adjuncts to avoid iatrogenic complications associated with increased morbidity and mortality.^(9)^

If lactate levels fail to decrease following optimization of these flow-related variables, the patient should be carefully reassessed for other (correctable) causes, for example, a previously unrecognized septic source, drug effects, tamponade, or bowel ischemia.^(1)^ In the absence of such causes, it is important to acknowledge that patients may still fail to improve despite ‘optimal’ conventional treatment with fluid, vasoactive agents, and mechanical organ supports. It remains unclear what, if anything, should be done in this situation beyond maintaining adequate perfusion, adequate source control, good patient care, and preventing iatrogenic harm.

## CONCLUSIONS

Patients with hyperlactatemia represent a medical emergency requiring the immediate creation of a clinical context to identify and treat the likely cause. As tissue hypoperfusion is common in such situations, enhancing tissue oxygen delivery to improve macro- and microcirculatory indicators of perfusion is an important first step. Careful consideration should be placed on the benefit-harm balance of continuing resuscitation in patients with normal perfusion parameters in whom hyperlactatemia persists. Such patients pose a clinical challenge, requiring reassessment of the diagnosis, including missed diagnoses, and consideration of possible additional treatments. In some cases, for example, established mitochondrial dysfunction, no clinical therapy yet exists that has been shown to improve outcomes. In this situation, good supportive care is the best that can be currently offered.

## Data Availability

Not applicable. It's a viewpoint.
